# Structure of unliganded membrane-proximal domains FN4-FN5-FN6 of DCC

**DOI:** 10.1007/s13238-017-0439-x

**Published:** 2017-06-29

**Authors:** Lorenzo I. Finci, Jie Zhang, Xiaqin Sun, Robert G. Smock, Rob Meijers, Yan Zhang, Junyu Xiao, Jia-huai Wang

**Affiliations:** 1 State Key Laboratory of Biomembrane and Membrane Biotechnology, College of Life Sciences, Peking University, Beijing, 100871 China; 2 Dana-Farber Cancer Institute, Harvard Medical School, Boston, MA 02215 USA; 3 European Molecular Biology Laboratory (EMBL), Hamburg Outstation, Hamburg, Germany; 4 PKU-IDG/McGovern Institute for Brain Research, Peking University, Beijing, 100871 China; 5 State Key Laboratory of Protein and Plant Gene Research, School of Life Sciences, and Peking-Tsinghua Center for Life Sciences, Peking University, Beijing, 100871 China; 6 Beijing Advanced Innovation Center for Structural Biology, Tsinghua-Peking Center for Life Sciences, School of Life Sciences and School of Medicine, Tsinghua University, Beijing, 100084 China; 7 The Francis Crick Institute, London, NW1 2BE UK

**Dear Editor,**

Deleted in colorectal cancer (DCC) is a single pass transmembrane glycoprotein that was originally identified in humans as a candidate tumor suppressor (Fearon et al., 1990). DCC belongs to the immunoglobulin superfamily, and the extracellular fragment is composed of four immunoglobulin-like (Ig-like) domains followed by six fibronectin type III (FN) domains. DCC plays a pivotal role in axon guidance by mediating a combination of attractive and repulsive effects through interactions with the diffusible guidance cue, netrin-1 (Hong et al., 1999) and draxin (Ahmed et al., 2011).

We have recently reported the crystal structure of DCC membrane-proximal domains FN5 and FN6 (DCC_FN56_) in complex with netrin-1. The structure revealed two distinct binding sites located between DCC_FN56_ and netrin-1 (Finci et al., 2014). Netrin-1 consists of a laminin-like domain (LN), followed by three EGF domains (EGF1, EGF2, and EGF3), and a C-terminal netrin-like domain (NTR) (Kennedy et al., 1994). The designated binding site 1 in our crystal structure is DCC-specific, exclusively involving the FN5 domain of DCC and the EGF3 domain of netrin-1, whereas the designated site 2 is a unique and highly conserved anion-dependent binding site that involves both the FN5 and the FN6 domains of DCC engaging the EGF1-EGF2 domains of netrin-1. Another structure determined in parallel utilized a similar netrin-1 construct, from chicken, along with a different DCC construct, from mouse, containing the FN4-FN5 domains (DCC_FN45_) (Xu et al., 2014). Both structures share the same binding site 1 via the FN5 domain and the EGF3 domain of netrin-1. In addition, their structure reveals another binding site on netrin-1’s LN domain interacting with the DCC FN4 domain, DCC_FN4_ (referred to here as binding site 0). The two structures are complementary to each other, encompassing FN4, FN5, and FN6 domains of DCC, interacting with the netrin-1 N-terminal LN domain and all three of the netrin-1 EGF domains (Finci et al., 2015).

Located between the functional DCC_FN4_ and DCC_FN5_ domains is an 8–28 amino acid linker, which is contingent on the particular DCC isoform. The DCC gene has two alternatively spliced isoforms. Cooper and colleagues demonstrated that the expression of alternatively spliced isoforms of mouse DCC is developmentally regulated. Their analyses on mRNA expression revealed that in D9.5 and D10.5 embryos, the DCC_short_ isoform (with 8-residue linker) was predominantly expressed, whereas between D10.5 and D11.5 of embryogenesis, the full-length DCC_long_ isoform (with 28-residue linker) mRNA was upregulated (Cooper et al., 1995). A recent study by Leggere et al. shows that the RNA-binding family protein NOVA controls the alternative splicing of DCC. They conclude that the two DCC isoforms are functionally distinct, and the Netrin-1/DCC_long_ interaction functions for axon outgrowth and guidance (Leggere et al., 2016). The linker must be important for the two isoforms to perform their function. An intriguing question yet to be addressed is how the 28-residue long flexible linker of an unliganded receptor eludes degradation in the extra-cellular environment? Here we have undertaken a structural and functional analysis of the DCC construct DCC_FN456,_ with a 22-residue long “artificial” linker composed of Ser-Gly-Gly (SGG) repeats (herein referred to as SGG-22) between the FN4 and FN5 domains. We have crystallized and analyzed the structure of this DCC_FN456_ construct. From the structure, we discuss how a consolidated FN5 can serve as the key netrin-1 binding domain, and how a closed configuration, where the FN4 domain “lies” on the rod-like FN5-FN6 domains, can be proposed as a resting state of the DCC receptor for stability.

It has been acknowledged that the FN4, FN5, and FN6 domains of the DCC receptor are all structurally and functionally required for netrin-1 binding (Finci et al., 2014; Xu et al., 2014). Another netrin-1 receptor, neogenin has a high sequence identity (around 46%) to that of DCC, and yet is most divergent in the FN4-FN5 linker region down to about 17% identity between the two receptors (Cooper et al., 1995). How does this long flexible and less homologous linker play its biological function? In order to dissect the relevance of the sequence and the length of the linker, we first utilized the cell-binding assay to probe the function of DCC_FN456_ with different linkers of different length between FN4 and FN5. The cell-binding assay has been previously utilized to describe interactions between DCC and netrin-1 in the binding site 1 and binding site 2 (Finci et al., 2014). We show here that when the linker was completely deleted, the binding was obliterated (ΔLinker4-5, in Fig. 1A). Next, we tested different constructs, replacing the wild-type linker with a sequence unrelated “artificial” linker composed of an increasing number of SGG repeats, designated as Linker4-5-nr. The DCC constructs with one to five SGG repeats exhibited an obliteration of function, whereas a linker of six to eight repeats exhibited a rescue of cell-binding, indicating a recovery of DCC function. Based on these results it appears that the cell-binding function of DCC is independent of the linker sequence, however is constrained by the length, which is consistent with the less sequence conserved linker region mentioned above in comparison to neogenin. A linker with 19 amino acid residues seems minimally required for separating the functional FN4 and FN5 domains in the context of cell-binding. To verify the difference in netrin-1 adhesion *in vitro* between the wild-type versions of the human long and short isoforms of DCC, an AVEXIS assay (Bushell et al., 2008; Gao et al., 2015) was then performed. A fragment of the long isoform of human DCC_FN456_ was inserted into the AVEXIS prey vector. The LN and EGF domains of human netrin-1 relevant for DCC-binding were inserted into the AVEXIS bait vector. Both proteins were expressed in HEK 293T cells, and binding was detected by measuring the absorbance of the nitrocefin substrate. For comparison, the relevant short isoform of human DCC was constructed by deleting residues 819–838, which lie within the linker (ΔLinker4-5). This construct shows significantly reduced binding to human netrin-1 (Fig. 1B). We therefore conclude that the DCC short isoform shows reduced netrin-1 binding, because it cannot use all possible combinations of the three DCC binding sites present on netrin-1.

**Figure 1:**
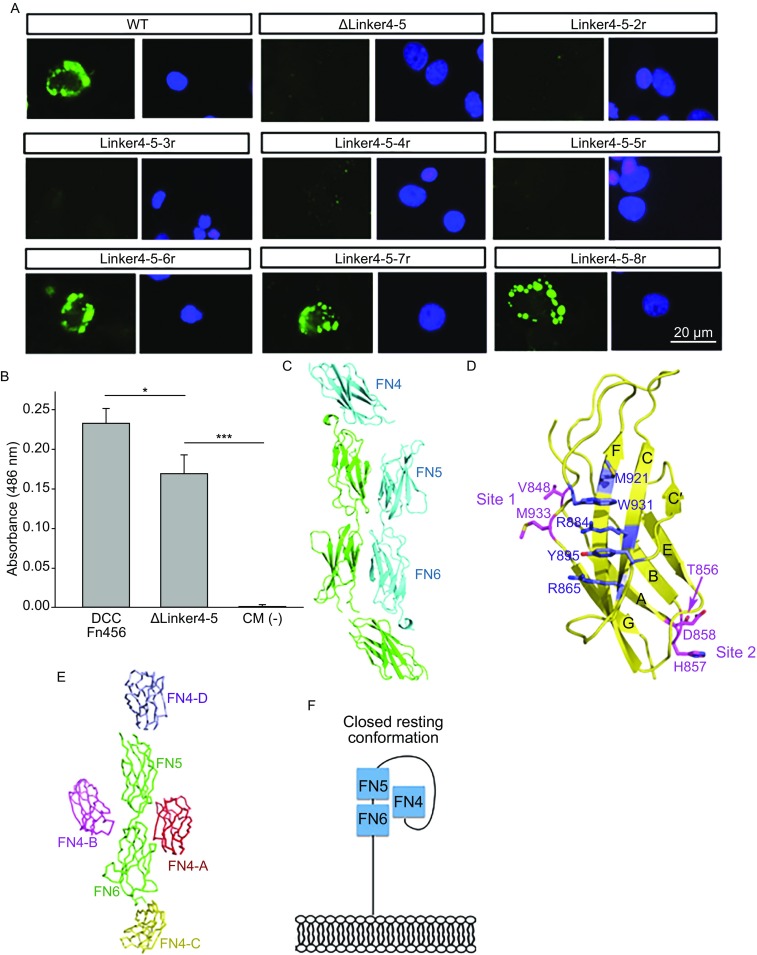
**Structure and functional analysis of DCC456 with artificial linker**. (A) The cell-binding assay was used to probe interactions between different constructs of DCC_FN456_ and netrin-1. Serine-glycine-glycine (SGG) repeats are represented by r. Constructs with no linker (Δlinker4-5) and 1 to 5 SGG repeats (such as linker4-5-5r) show no function, whereas 6 to 8 SGG repeats show function. Netrin-1 binding to COS cells transfected with DCC constructs is detected with a netrin-1 antibody (Abcam, #ab78854) and a secondary green fluorescent antibody. The cell nuclei were indicated by blue DAPI staining. Scale bar: 20 μm. (B) The *in vitro* binding of human DCC ectodomains and netrin-1s was examined using an Avexis assay (Bushell et al., 2008; Gao et al., 2015). Briefly, netrin was biotinylated and attached to a streptavidin plate. Binding was monitored by the colorimetric conversion of nitrocerfin by a β-lactamase fusion to DCC. In the absence of cellular membrane components, binding was observed between both the DCC_FN456_ and the ΔLinker4-5 to netrin. However, binding was significantly diminished for the ΔLinker4-5 construct. CM = conditioned media in which DCC was not expressed. *n* = 3, *P < 0.05, ***P < 0.001, error bars indicate s.d. (C) The crystal structure of DCC_FN456_ has 2 molecules in the asymmetric unit. (D) Hydrophobic ladder on the CFG β-sheet of the FN5 domain. The FN5 domain has a hydrophobic ladder that is formed from a group of hydrophobic residues M921, W931, Y895 and the aliphatic portion of side chains from R884 and R865 from different β-strands that stack upon one another to stabilize the structure. Critical residues involved in netrin-1 binding are positioned on either side of this structural feature, shown as site 1 and 2, respectively. (E) Crystal packing of DCC_FN456_. Four FN4 domains pack around one FN5-FN6 entity in DCC_FN456_ crystal lattice. (F) Proposed model of closed “resting” state of DCC_FN456_

The DCC construct that was expressed in *E*. *coli* and crystallized contained a linker of 22 amino acids, and the linker is represented as G(SGG)7. The P2_1_ crystal structure was determined at 3.0 Å resolution with an *R*_free_/*R* = 0.223/0.278 via molecular replacement. The search models used were the DCC_FN56_ domain (PDB 4URT) (Finci et al., 2014), and the DCC_FN4_ domain (PDB 4PLO) (Xu et al., 2014). There are two molecules in one asymmetric unit, depicted in Fig. 1C. Since there is a 22-residue linker between the FN4 and FN5 domains, which is disordered in the crystal structure and can potentially stretch somewhere around 80 Å, we cannot authenticate which FN4 domain is linked to which FN5 domain to form one molecule. As will be discussed later, the relative position of the blue-colored FN4 domain with the blue-colored rod-like FN5-FN6 domains shown in Fig. 1C is just one possibility.

The crystal structure DCC_FN456_ presented here, represents three tandem FN domains. FN domains are domains that are evolutionarily conserved and consist of roughly 100 amino acids. Along with other domains, such as the Ig-like domains, the epidermal growth factor (EGF) domains, they form modular structures as components of cell surface receptors that function in the immune system as well as the nervous system. Unlike Ig-like domains though, FN domains do not have disulfide bonds between the two opposing β sheets. Nevertheless, the FN domains have an invariant tryptophan at the center of the β strand B in place of the cysteine in the Ig-like domain that assists to form a hydrophobic core to stabilize the FN domain. Still, compared to the Ig-like domains, the FN domain is less robust and may be subject to mechanical deformation (Rounsevell and Clarke, 2004). For this reason, we observe a uniquely stabilizing structural feature for the key netrin-1-binding domain FN5 of DCC. In the concave-shaped CFG β sheet, a group of hydrophobic residues M921, W931, Y895 and the aliphatic portion of side chains from R884 and R865 emanated from different strands stack on top of one another, forming a “hydrophobic ladder” (Fig. 1D). These interactions should substantially increase the stability of the FN5 domain to resist any applied force. The critical residues involved in the netrin-1 binding to site 1 and site 2 (Finci et al., 2014) cluster on either side of this hydrophobic ladder, respectively (Fig. 1D). A stable, solid FN5 domain has therefore evolved to serve a key role in netrin-1 binding, able to resist the force generated by the binding from the either side. We have previously seen that one rigid guidance cue molecule, netrin-1, binds two DCC receptors (Finci et al., 2014). Now it is apparent that one stable, solid FN5 can mediate two separate netrin-1 interactions, which is consistent with the previously proposed netrin-1/DCC clustering model (Finci et al., 2015)

A high proportion of cell-surface receptors are modular. Usually, there will be just a couple of residues in between domains to provide some flexibility to the receptors. However, there are also cases where a long enough linker between domains exists, like the one between the FN4 and FN5 domains of DCC. Apparently, this kind of long linker is prone to be cleaved in the extra-cellular environment. If such a long linker does specifically serve a function, there might likely be a way to protect it from cleavage in the unliganded state. One previously reported example is found in the human fibronectin, where there is a 21-residue linker between its first and second type III FN domains (Vakonakis et al., 2007). In the “resting” state, the FN molecule is in a stable and “closed” conformation with ^1^FNIII and ^2^FNIII folded into a weak but specific contact that makes the molecule soluble. At the initial step of fibrillogenesis the closed ^1^FNIII and ^2^FNIII opens up and the long linker stretches out for functional purposes. We propose that a similar scenario might be applicable for the long linker between FN4 and FN5 (also type III fibronectin domains) of DCC, which has ten domains in tandem located on the cell surface.

In the crystal lattice of the structure we determined, four FN4 domains are positioned around one rod-like FN5-FN6 entity (Fig. 1E). These four FN4 domains should belong to four different DCC_FN456_ molecules in the crystal, respectively. As described above, the 22-residue linker between FN4 and FN5 in the current structure of DCC_FN456_ is disordered, so we do not have direct evidence on which one of the four FN4 domains should be linked to the particular FN5-FN6 entity to form one DCC_FN456_ molecule in Fig. 1E. Table 1 lists the surface buried area for each of these four FN4 domains to contact the FN5-FN6 entity shown in Fig. 1E. It is clear that domain FN4-A makes the largest contacts with the FN5-FN6 entity. An early statistic on specific versus non-specific protein-protein contacts observed in protein crystals demonstrated that a functionally relevant interface should at least have more than 700 Å^2^ buried surface area, and a significantly smaller interface is likely generated from crystal packing artifacts (Janin, 1997). Our crystal structure indicates some specific interactions between the bottom portion of the FN4-A and the FN6, including a hydrogen bond positioned between E787 of FN4-A and S995 of FN6 and between K970 of FN6 to the main chain carbonyl group of E784 of FN4-A, etc. (Supplementary Figure). We can thus envision that this FN4-A domain more likely connects with the FN5-FN6 entity as shown in Fig. 1F to form one DCC_FN456_ molecule. The distance between the C-terminus of FN4-A and the N-terminus of FN5 is around 57 Å, which is appropriate for a 22-residue linker to connect to. We then would like to propose that this configuration of FN4-A-linker-FN5-FN6 molecule might represent a stable “resting” state of the DCC receptor. Compared with the short 8-residue linker of the DCC isoform, a full-length 28-residue linker DCC isoform was upregulated in late stage of embryogenesis, particularly in the central and peripheral nervous development (Cooper et al., 1995). This 28-residue DCC isoform could assume this FN4-A-linker-FN5-FN6 closed resting conformation for increased stability. When netrin-1 comes to bind to DCC, the FN4-A-linker-FN5-FN6 molecule could assume an open extended conformation to interact with binding site 0 and binding site 1, and further propagate into a cluster to trigger axon attraction signaling.

**Table 1:** The surface buried area of the four DCC FN4 domains on FN56

FN4-A	1,106 Å^2^
FN4-B	144 Å^2^
FN4-C	748 Å^2^
FN4-D	195 Å^2^

Note: Out of the 4 potential FN4 domains that pack around FN56, the FN4-A has the largest surface buried area indicating that it makes the largest contact with FN56

## Electronic supplementary material

Below is the link to the electronic supplementary material.

Supplementary material 1 (PDF 288 kb)
