# Supplementary material for: Structure of unliganded membrane-proximal domains FN4-FN5-FN6 of DCC
Source: Protein Cell. 2017 Jun 29;8(9):701–5. doi: 10.1007/s13238-017-0439-x (PMC5563286; doi:10.1007/s13238-017-0439-x)

Supplementary Methods and Materials:
DCC_{FN456} preparation

DCC Fibronectin domains 4, 5 and 6 including a 22 amino acid artificial-linker between domains 4 and 5 (DCC_{FN456}) from *Homo sapiens* were cloned into the pET-21a vector, with a C-terminal Strep-tag and a C-terminal His_6-tag. The recombinant plasmid was transformed into BL21 (DE3) competent cells. For each preparation, the transformed cells were induced with 0.01 mM IPTG for 20 hours at 16℃ when the OD_{600} was approximately 0.8. The cells were harvested and re-suspended in 100 mL lysis buffer (100 mM Tris pH 8.0, 150 mM NaCl) and lysed by sonication. Following ultra-centrifugation at 10,000 rpm for 30 minutes, the protein was purified *via* the Strep-tag, dialyzed in PBS, and then purified *via* the His-tag. The remaining impurities and degradation product were removed with size exclusion chromatography (S-75). The protein preparations produced 9 mg of total protein. Prescission Protease (Genescript) was utilized to cleave the affinity tags. To remove the protease we used a GST column, and then a Strep-Tactin column to remove the uncleaved DCC_{FN456}. The final sample was dialyzed against 150 mM NaCl, 100 mM Tris-HCl, at pH 8 and concentrated to 16 mg/mL.

Crystallography of DCC_{FN456}

DCC_{FN456} was set up for crystallization screening using a 0.5 l sample to 0.5 l crystallization buffer. Standard crystallization screens, including Index, PEG Ion, and Crystal Screen I and II (Hampton Research) were then set up. Crystals appeared in condition 21 from Hampton Crystal Screen 2 that contained 0.1 M Sodium Phosphate Monobasic Monohydrate, 0.1 Potassium Phosphate Monobasic, 0.1 MES Monohydrate pH 6.5, 2.0 M Sodium Chloride. The crystals diffracted poorly to about 6Å. Crystals from this droplet were used in subsequent seeding experiments using the SeedBead kit (Hampton). Diffraction quality crystals appeared in 6 months as larger rods assembled in a sea urchin fashion. Single crystals were looped and placed in a drop containing the mother liquid supplemented with 20% glycerol for cryo-protection and flash frozen in liquid nitrogen. Data was collected at the Photon Factory (KEK) on beamline BL-1A, Japan, with a Pilatus 2M-F detector and processed with HKL2000 (HKL Research). The structure of DCC_{FN456} was determined by molecular replacement using the DCC_{FN4} and DCC_{FN56} structures in Phaser (McCoy et al., 2007). The structural model was manually adjusted in Coot (Emsley and Cowtan, 2004) and refined using Phenix (Murshudov et al., 1997). Five percent of randomly selected reflections were used for cross-validation (Brunger et al., 1998). Data statistics are shown in Supplementary Table.
The structural data has been deposited in Protein Data Bank with the code: 5X83

Cell Binding Assays

Cell binding assays were performed as described previously (Finci et al., 2014) using COS cells transfected with wild-type or mutant DCC constructs, which were then incubated in the media containing 10 mg/mL netrin-1.

AVEXIS assays

Human netrin-1s (residues 25-453) was cloned into the Avexis bait vector and human DCC Fn456 (residues 719-1098) was cloned into the Avexis prey vector (Bushell et al., 2008). ΔLinker4-5 was identical to DCC Fn456 except with internal deletion of residues 819-838. The Avexis assay was performed as previously described (Gao et al., 2015). Briefly, proteins were transiently transfected in HEK293 and grown in suspension using Freestyle medium (Gibco) supplemented with 1% FBS. The collected supernatants were dialyzed in HBS (10 mM Hepes, 140 mM NaCl, 5 mM KCl, 2 mM CaCl2, 1 mM MgCl2, pH 7.4). Empty prey vector was also used to produce conditioned media as negative control. Netrin bait expression was verified by Western blot. Prey protein concentrations were normalized via calorimetric reaction of 0.1 mg/ml nitrocefin (Calbiochem) in a microplate and measurement of absorbance after 20 minutes (486 nM, Tecan). For the binding assay, a streptavidin-coated 96-well plate (Nunc) was blocked in 100 μL HBS with 2% BSA, coated with 50 μL netrin bait, and incubated with 50 μL normalized prey, with three washes after each step using 100 μL HBS and 0.05% Tween-20. 50 μL 0.1 mg/ml nitrocefin was incubated in wells for 20 minutes before absorbance measurement (486 nm, Tecan). Blank subtraction was performed using wells in which netrin prey was replaced with HBS buffer.

Supplementary References

Brunger, A.T., Adams, P.D., Clore, G.M., DeLano, W.L., Gros, P., Grosse-Kunstleve, R.W., Jiang, J.S., Kuszewski, J., Nilges, M., Pannu, N.S., *et al.* (1998). Crystallography & NMR system: A new software suite for macromolecular structure determination. Acta Crystallogr D Biol Crystallogr *54*, 905-921.

Emsley, P., and Cowtan, K. (2004). Coot: model-building tools for molecular graphics. Acta Crystallogr D Biol Crystallogr *60*, 2126-2132.

McCoy, A.J., Grosse-Kunstleve, R.W., Adams, P.D., Winn, M.D., Storoni, L.C., and Read, R.J. (2007). Phaser crystallographic software. J Appl Crystallogr *40*, 658-674.

Murshudov, G.N., Vagin, A.A., and Dodson, E.J. (1997). Refinement of macromolecular structures by the maximum-likelihood method. Acta Crystallogr D Biol Crystallogr *53*, 240-255.

Supplementary Table. Data Collection Statistics

Data Collection Statistics	DCC_{FN456} Artificial Linker
Resolution (last shell) (Å)	42.1-3.0 (3.05-3.0)
X-ray Source	KEK BL-1A Pilatus 2M-F
Wavelength (Å)	1.10000
Space group	$P2_1$
Cell dimensions<br>*a, b, c* (Å)<br>α, β, γ (°)	 53.5, 127.0, 57.2<br>90.0, 99.7, 90.0
Reflections Observed	14709
Completeness (%)	97.4 (100)
R_{pim}	18.8 (71.0)
I/ *I*	8.6 (2.2)
Redundancy	6.0 (5.9)
Wilson B-factor	49.3
Refinement Statistics	
No. of protein atoms	4,627
R_{work}/R_{free} (%)	22.3/27.8
Resolution (Å)	3.0
Average B-factor ($Å^2$)	55.6
RMSD Bond length (Å) / angles	0.003 / 0.64
Ramachandran Statistics	
Favored	95.3%
Allowed	4.7%
Outliers	0%

The numbers in parenthesis are for the highest resolution bin

$R_{p.i.m} = \sum_{hkl} [1/(N-1)]^{1/2} \sum_i |I_i(hkl) - \overline{I(hkl)}| \Big/ \sum_{hkl}\sum_i I_i(hkl)$

Supplementary Figure. Interactions between FN4 and FN6

Hydrogen-bonding interactions between FN4 (red) and FN6 (turquoise) are depicted. Two specific hydrogen bonds positioned between E787 of FN4-A and S995 of FN6 and between K970 of FN6 to the main chain carbonyl group of E784 of FN4 are highlighted.

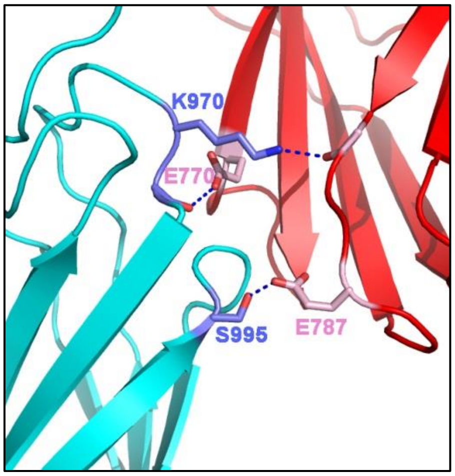

Supplement: Supplementary file 1 — Supplementary material 1 (PDF 288 kb) [file 13238_2017_439_MOESM1_ESM.pdf]
